# Development of a Consistent and Reproducible Porcine Scald Burn Model

**DOI:** 10.1371/journal.pone.0162888

**Published:** 2016-09-09

**Authors:** Christine J. Andrews, Margit Kempf, Roy Kimble, Leila Cuttle

**Affiliations:** 1 Centre for Children’s Burns and Trauma Research, The University of Queensland, Centre for Children’s Health Research, South Brisbane, Queensland, Australia; 2 Centre for Children’s Burns and Trauma Research, Queensland University of Technology, School of Biomedical Sciences, Institute of Health and Biomedical Innovation at Centre for Children's Health Research, South Brisbane, Queensland, Australia; Michigan State University, UNITED STATES

## Abstract

There are very few porcine burn models that replicate scald injuries similar to those encountered by children. We have developed a robust porcine burn model capable of creating reproducible scald burns for a wide range of burn conditions. The study was conducted with juvenile Large White pigs, creating replicates of burn combinations; 50°C for 1, 2, 5 and 10 minutes and 60°C, 70°C, 80°C and 90°C for 5 seconds. Visual wound examination, biopsies and Laser Doppler Imaging were performed at 1, 24 hours and at 3 and 7 days post-burn. A consistent water temperature was maintained within the scald device for long durations (49.8 ± 0.1°C when set at 50°C). The macroscopic and histologic appearance was consistent between replicates of burn conditions. For 50°C water, 10 minute duration burns showed significantly deeper tissue injury than all shorter durations at 24 hours post-burn (p ≤ 0.0001), with damage seen to increase until day 3 post-burn. For 5 second duration burns, by day 7 post-burn the 80°C and 90°C scalds had damage detected significantly deeper in the tissue than the 70°C scalds (p ≤ 0.001). A reliable and safe model of porcine scald burn injury has been successfully developed. The novel apparatus with continually refreshed water improves consistency of scald creation for long exposure times. This model allows the pathophysiology of scald burn wound creation and progression to be examined.

## 1 Introduction

Burns are a common and potentially devastating cause of injury in childhood. Scald burns as a mechanism of thermal injury are of particular importance as globally they are still the most commonly treated paediatric burn injury [1]. In Australia approximately 2000 children will be hospitalized each year with burns, with over half of these admissions being for scalds (hot liquid or steam) [2–4]. Many experimental animal models of burn injury are reported in the literature (reviewed in [5, 6]) using rats, mice, rabbits, guinea-pigs and pigs with various mechanisms of thermal injury such as scald, contact, radiant heat and flame. Given the anatomical and physiological similarities between porcine and human skin [7–10], pigs are considered by many to be the optimal species for cutaneous thermal injury investigations [6, 8, 9, 11]. A porcine scald burn model is arguably the model of most translational relevance to paediatric burns as it is recognised clinically that the pathophysiology of scald burns is different to that of contact burns [12, 13]. However, whilst there are many published porcine contact burn models [10, 14–20], there are few porcine models that replicate a true scald injury such as those encountered by children.

Existing porcine scald burn models described by Henze *et al*.[21] and Radke *et al*.[22] used an immersion technique to create one large scald burn covering 30% of the total body surface area (TBSA). Although water is maintained at a consistent temperature for long durations, these studies are limited by testing only one temperature/duration combination per animal and the difficult logistics of suspending a large animal over a water bath. Brans *et al*.[12] utilised a bottomless applicator to create several small burns on the flank of the same animal. However, using a single application of water at the desired temperature (80°C for 10, 20, 30, 40 sec) is limited, as water rapidly cools in the device once removed from the heat source. To test for a broad range of burn conditions, the ability to create several small uniform wounds on the same animal is desirable. Additionally, maintaining a consistent water temperature within the scald device improves the consistency and reproducibility of scald burn creation.

Scald spill/splash injuries occur from exposure to very hot liquids for only a short skin contact duration e.g. hot beverage scalds. However, immersion scalds are also a frequent mechanism of scald burn injury, occurring as a result of exposure to more moderate temperature liquids for longer durations e.g. bath water scalds. The aim of this study was to develop a porcine scald model that could be used to examine clinically relevant burn conditions. Based on the design of Moritz and Henriques published studies [11, 23] a novel scalding device was developed. The apparatus allows for continuous refreshment of the hot water within the device and a more consistent water temperature for long durations. Additionally, in order to better understand how scald burns progress in the acute peri-burn period, it was desirable to create a burn area large enough such that multiple, well-spaced sequential biopsies could be taken from the same burn. Therefore, while Moritz and Henriques [11] created numerous (approximately 34) very small burns (4.9cm^2^) on each pig, our intent was to develop a model where several (8) moderately sized burns (16cm^2^) could be created on the same pig.

Several techniques were used in this study to analyse, compare and evaluate the wounds, validating the model’s ability to create reproducible and consistent burns. Evaluations included; subdermal temperature monitoring; quantitative histological analysis of tissue injury and Laser Doppler Imaging (LDI). Importantly, sequential analysis of the burns over 7 days allows for assessment of how the burn wounds evolve in the early post-burn period.

The primary focus of this manuscript is to provide a comprehensive description of a novel method for reproducible scald burn generation, exploring the extent of tissue injury resulting from exposure to a range of clinically relevant burn conditions. The model’s unique ability to replicate burn injury for a broad range of scald burn conditions is highly desirable and has wide application for researchers investigating novel burn therapies or diagnostics, as well as those investigating the pathology of burn injury.

## 2 Materials and Methods

### 2.1 Ethics Statement

All methods conformed to the Australian Code of Practice for the Care and Use of Animals for Scientific Purposes (7^th^ Edition) published by the Australian National Health and Medical Research Council. Ethics approval was obtained from the University of Queensland Animal Ethics Committee (Approval numbers: QCMRI/RCH/326/12/QCMRI/NHMRC and QCMRI/446/15/QCHF). All procedures were performed under a general anaesthetic and all efforts were made to minimise suffering.

### 2.2 Animals

Female Large White juvenile pigs of 27kg (approximately 12 weeks of age) were used for the study. Pigs were delivered to the animal house 7 days prior to commencing the experiment to allow for acclimatisation. Animals were given a standard pellet diet and free access to water. On days prior to procedures requiring administration of anaesthesia the pigs were fasted overnight.

### 2.3 General Anaesthetic and Monitoring

Anaesthesia was induced intramuscularly with Ketamine 13mg/kg (Ketamine 100mg/ml Ceva^™^, Glenorie, NSW, Australia) and 2mg/kg Xylazine (Ilium Xylazil 100mg/ml, Ilium^™^, Troy laboratories Pty Ltd, NSW, Australia). A size 4 laryngeal mask airway was inserted and anaesthesia was maintained at a surgical plane with 1%-2.5% Isoflurane (Attane, Bayer, Australia Ltd) in oxygen (1–3litre/min flow). Buprenorphine 0.01mg/kg (Temgesic^®^ 0.3mg/ml Reckitt Benckiser, Healthcare, UK) was administered intramuscularly peri and post-operatively to provide analgesia. A 24 gauge IV cannula was placed in the lateral auricular vein and intravenous fluids (0.9%NaCl) were administered at a rate of 5ml/kg/hr throughout the procedure. A transdermal Fentanyl 50μg/hr patch (Durogesic^®^50, Janssen-Lilag Pty Ltd, North Ryde, Australia) was applied for additional post-operative analgesia. Animals underwent the same general anaesthetic regime at each biopsy collection time point. On day 7 the animals were euthanased with 15ml of sodium pentobarbitone (Lethabarb^™,^, Virbac Pty Ltd, NSW, Australia) administered intravenously.

### 2.4 Wound creation

#### 2.4.1 Device

**Fig 1:**
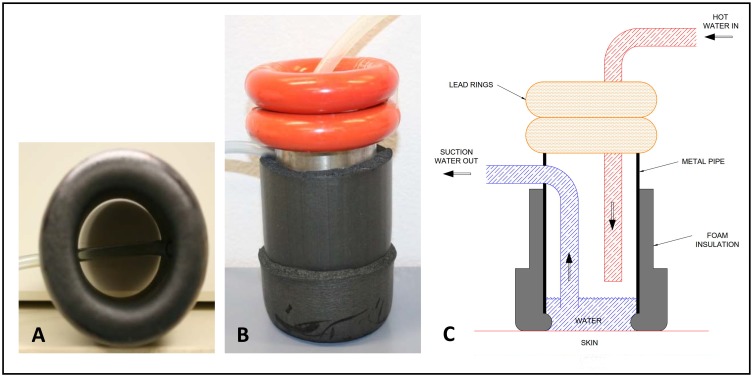
Scald creation device. Consists of metal pipe covered with insulation tubing which is folded onto itself at the bottom and a clear plastic vacuum suction tube. A. ‘padded lip’ bottom of device, B. side view of device with weighted rings placed on top, C. Schematic of device showing inflow and outflow of hot water.

The materials used to make the scald burn device can be readily sourced from hardware and plumbing stores available in most countries. The device consisted of a 60mm diameter stainless steel pipe with foam insulation tubing fitted around the outside to allow an operator to hold the device safely (Fig 1) The insulation material was folded back onto itself on the bottom edge to create a padded lip which was placed onto the animal (there was no direct contact between the metal pipe and the skin surface). The aperture at the bottom of the device for direct contact between water and the skin surface was approximately 50mm. To provide uniform downward pressure, two weighted lead rings (1kg when taped together) were placed on top of the device. Plastic tubing (4mm diameter) sitting 20mm above the bottom of the device was connected to vacuum suction, ensuring a consistent level of water was maintained within the device at all times.

A temperature controlled water bath (Grant Instruments, Cambridge, UK) was used to maintain the water at a constant, tightly regulated temperature. A digital 5411 Fluke^®^ thermometer (Fluke Australia Pty Ltd., North Melbourne, Australia) was used to measure the water temperature within the scald device. Water was pumped into the scald device at a rate of 1.3 litres per min using a submersible aquarium pump (set at 4.5Volts) and vacuum suctioned out at an equivalent rate. Using the maximum temperature values recorded from initial experiments and trials of the system external to the pig as a guide, we found that setting the water bath to 3°C higher than the desired target temperature (for temperatures ≥ 70°C) ensured the water in the device at the time of scald creation was most precise. The heat loss within the system for lower temperature burns was minimal and with experience we found setting the water bath to 0.3°C higher than the target temperature (for temperatures ≤ 60°C) was desirable. Pre-warming the device immediately prior to scalding and insulating the inflow tubing was also beneficial.

#### 2.4.2 Site of burn

In total eight burns were created on the thoracic paravertebral area of each animal (four on each side), with 2–3 centimetres between burns. Two replicate burns were created for each of four time/temperature combinations per pig. To account for any variation in healing due to anatomical location [24], burn positions on one side of the animal were assigned randomly and replicate burns (on the other flank) were assigned to the opposite anatomical location.

### 2.5 Scald burn creation

**Fig 2:**
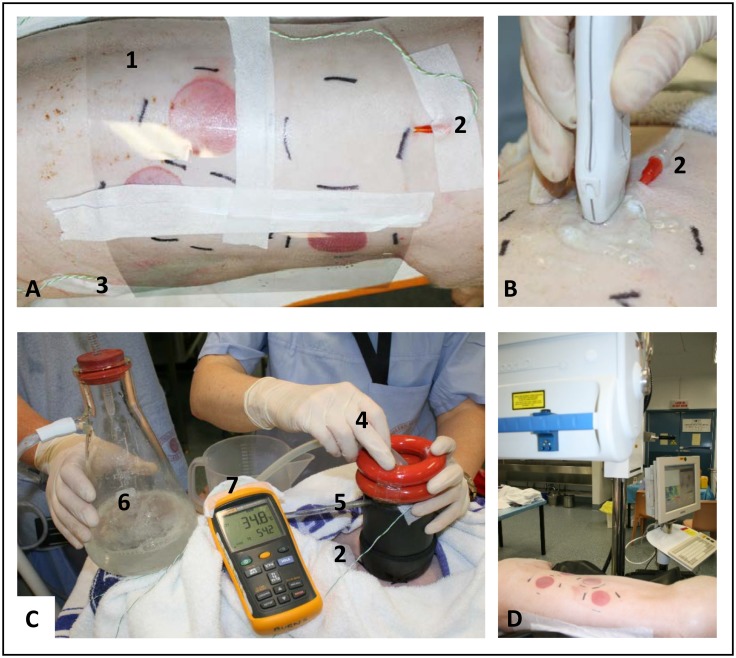
Scald burn creation and evaluation. A. preparation of the site for scalding, B. ultrasound assessment of subdermal temperature probe depth prior to scalding, C. scald burn creation and D. post-burn evaluation with Laser Doppler Imaging. 1. Shows protective plastic covering surrounding non-burn areas. 2. Subdermal temperature probe. 3. External skin temperature probe 4. Hot water inflow. 5. Suction water out. 6. Suction collection flask. 7. Fluke thermometer.

Preparation of the burn sites included clipping the hair on the back and flanks and marking the sites for burning using the device as a template. Protective clear plastic sheets were taped in place (Fig 2A1) to cover the immediate surrounding area and reduce the risk of an unintentional burn. Immediately prior to scalding, the device and tubing was pre-warmed for at least 1 min by running the system external to the pig. After pre-warming, the device was placed perpendicular to the skin surface and weighted rings were placed on top to provide uniform downward pressure for a leak proof seal. The same operator supported the device with one hand and controlled the hot water inflow tube with their other hand (Fig 2C). At the end of the exposure the inflow of hot water was halted by pinching the tube, the whole scald device was lifted off the animal and any excess water was quickly mopped up with a towel. A range of burn conditions were tested; 50°C for 1, 2, 5 and 10 min; and 60, 70, 80 and 90°C for 5 sec.

After wound assessments (detailed below), all burns were dressed with Melolin^™^ and Fixomull^®^ (Smith & Nephew, Australia) which was changed at each time point. To further protect the burn area, custom made garments [15] were fitted.

### 2.6 Temperature monitoring

Body temperature was measured at regular intervals using a rectal digital thermometer (MC-110B, Omron Corporation, Japan). Subdermal temperature probes (K type thermocouples, Radiospares Components Pty LTD., Smithfield, Australia) were used to monitor the temperature within the skin during scald creation, using our previously published method [25–27] (Fig 2A2). The depth of the probe in the subcutaneous tissue was measured using ultrasound (LOGIQ e R7 series with a 22 MHz hockey stick probe (GE Healthcare)) (Fig 2B). Temperatures were logged by the Fluke thermometer every second once the heated water was applied or every 10 sec for burns of 10 min duration. Logging of subdermal temperatures was continued for at least 60 sec after the heat source was removed, or until a maximum temperature was reached. The external skin surface temperature was measured at a neighbouring site, using a separate temperature probe (Fig 2A3).

### 2.7 Wound Assessment

After burn creation and at each biopsy time point, wounds were examined visually and the total area of the burns were assessed using a Visitrak^™^ device (Smith & Nephew, Australia) [15]. Digital photographs were also taken of each wound using a Canon EOS 300D digital SLR camera (Canon Australia, North Ryde, Sydney).

#### 2.7.1 Histological investigation

**Fig 3:**
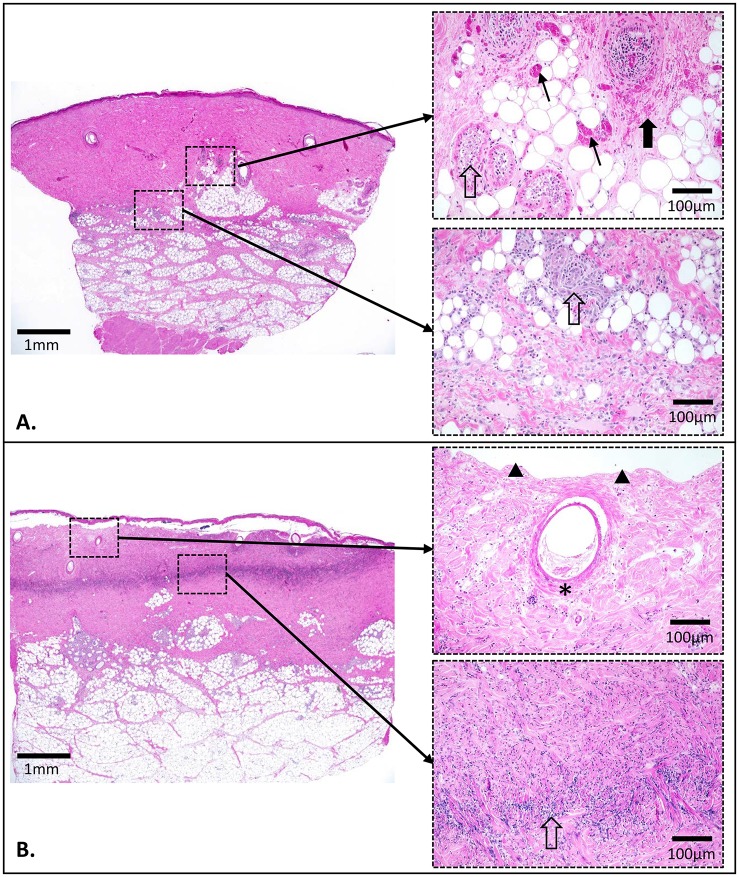
Examples of tissue injury markers identified using H & E staining. Images from A. 90°C for 5 sec and B. 50°C for 10 min scald at 72hrs post-burn. Thick black arrow indicates free red blood cells, thin black arrow indicates blocked vessels, black triangles show where the epidermis has lost adherence, asterisk indicates adnexal necrosis and open black arrow points to inflammatory cell infiltration seen; within glands, perivascular and as a thick band within the tissue of the dermis.

Full thickness 8mm skin biopsies were obtained from all of the burns at 1hr, 24hr and 3days and 7days post-burn. A sample of normal skin from each animal was also taken at each time point. To minimise any local inflammatory effects post-biopsy, sequential biopsies were taken from separate representative areas. Each biopsy sample was fixed in 10% neutral buffered formalin for 24hrs and embedded in paraffin. Routine haematoxylin and eosin (H&E) staining was performed on 5μm thick paraffin sections. Sections were digitally captured using a Nikon EP600 microscope (Nikon Instruments Inc, USA) fitted with a Spot RT slider cooled CCD camera (SPOT Imaging Solutions^™^, Sterling Heights, USA) and scored by an examiner blinded to burn conditions. Quantitative measurements (in mm) for the minimum, maximum and average thickness of the dermis and depth of dermal damage over the entire section were electronically calculated using Image Pro Plus v.5.1 software (Media Cybernetics, Silver Spring, USA). All dermal elements were evaluated for damage, with a line of damage representative of the deepest tissue injury detected for each section. The average depth of this line was used to calculate results presented here as % injury to dermis by depth (average depth of damage to dermis divided by the average total depth of the dermis). Markers of tissue injury for H&E sections included; blocked vessels[28], endothelial cell injury[29], adnexal necrosis[30], infiltration of inflammatory cells and dilation of lymphatic vessels[16] (Fig 3).

### 2.8 Laser Doppler Imaging

Laser Doppler Imaging (LDI) was performed at each time point to assess blood flow to the wound. The LDI scanner (Moor LDI 2, Moor Instruments, Devon, UK) was positioned perpendicularly over the flank of the animal such that all four burns were imaged simultaneously (Fig 2D). The average distance between the scanner head and the skin surface was 37cm. Scans were performed using the settings for a large scanning area with high resolution (256 X 256 pixels) and a slow scan speed of 4ms/pixel. Analysis of images using Moor LDI Image Processing v2.4 (Moor Instruments Ltd, Devon, UK) was performed by defining the wound area as a ‘‘region of interest”(ROI) for each burn and calculating the average perfusion units (PU) per wound. Individual biopsy sites within each burn were also traced as an ROI and excluded from calculations. For each flank one perilesional ROI of normal skin was measured as a control. The mean blood perfusion ratio of burned skin (B) to normal (N) perilesional skin was calculated as described previously by others [31]. A B/N ratio <1 indicates less blood perfusion than normal skin, = 1 designates same perfusion as normal, >1 shows increased perfusion from normal. Burn areas showing no macroscopically visible signs of injury (at the time of scanning) were not reviewed and blood perfusion was considered the same as normal skin (B/N = 1). Higher than normal blood flow indicates a more superficial burn and low flow indicates a full thickness burn, whereas deep partial thickness dermal burns may have a blood flow similar to or lower than normal skin.

### 2.9 Data analysis

The statistical analysis was performed using GraphPad Prism V6 (GraphPad Software, Inc. California, USA) and SPSS 22 (IBM Corporation, Armonk, N.Y, USA). Results are reported as mean ± standard error of the mean (SEM) unless otherwise stated. Analysis of the LDI ratios was performed using a method previously reported [31]. A two way analysis of variance (ANOVA) was performed at each observation time point with a Tukey’s multiple comparison test to compare the burn conditions for significant differences (p<0.05). A Spearman’s correlation was used to calculate an intra-observer reliability rating for histological scoring of dermal damage, with significance set at p = 0.01(2 tailed).

## 3 Results

A total of 40 individual burns were included for analysis. There were n ≥ 4 replicates of each burn temperature/time combination (50°C for 1, 2, 5 and 10 min and 60°C, 70°C, 80°C and 90°C for 5 sec). The average burn size was 16.36 ± 1.59cm², with a length of 4.79 ± 0.32cm and a width of 4.52 ± 0.35cm. The pig’s average weight on scald creation day was 27kg and the estimated total body surface area of the burns was 2% [32]. The average thickness of the dermis on scald creation day was evaluated with histology to be 2.29 ± 0.45mm.

### 3.1 Validation of water temperature in scald device

**Fig 4:**
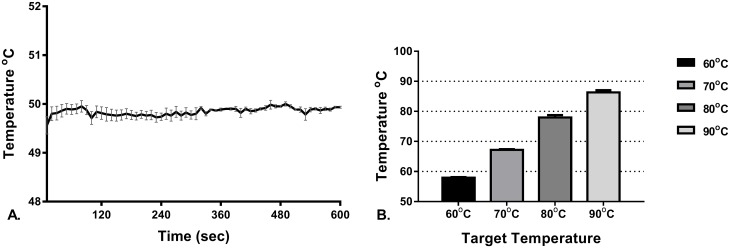
Temperature of water in the device during scald creation. A. 50°C water for 5 and 10 min exposure (n = 8). B. 5 second duration exposures showing the maximum temperature recorded for water in the scald device for each target temperature (n ≥ 4 for each temperature). Bars are mean ± SEM.

Temperatures logged from the probe within the scald device fluctuated in the first 5–10 seconds before stabilising, at which point a more consistent and accurate reading of the water temperature was obtained. The temperature of the water in the scald device when the input water temperature was 50°C (from the water bath) was 49.8°C ± 0.1°C (Fig 4A). The maximum temperature recorded for water in the device for the 5 sec duration scalds is shown in Fig 4B.

### 3.2 Consistency of scald burn creation

#### 3.2.1 Macroscopic

**Fig 5:**
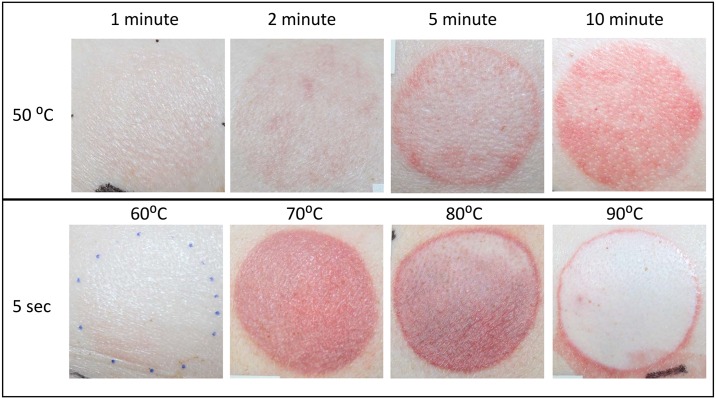
Wound appearance 1 hr post-burn. Intensity of colour varies with burn severity.

All burns had a uniform circular shape with a consistent red colour throughout the burn area immediately after creation. By 1 hr post-burn the more superficial burns were beginning to lose their intensity, becoming less distinct with a mottled white and red appearance, while the distinctive deep red/hyperaemic appearance of the more intermediate severity burns had intensified (Fig 5). At 1 hr, the 90°C for 5 sec scalds were observed to have a red hyperaemic ring around the burn with a uniform blanched white appearance, indicative of a more severe burn.

#### 3.2.2 Microscopic

**Fig 6:**
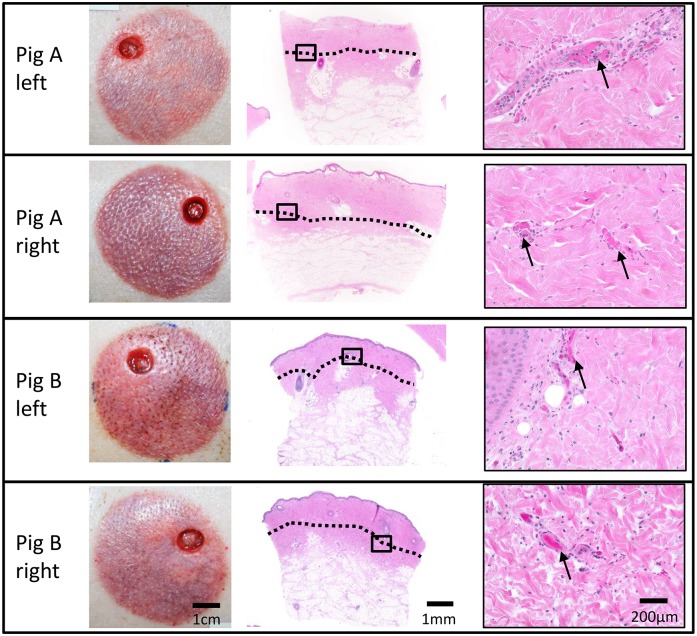
Comparison of macroscopic and histologic appearance for 4 replicates of 50°C/10 min burns at 24hrs post-burn shows consistency in appearance and depth of injury. Dotted black line represents the deepest level where tissue injury was observed. Black rectangle indicates location of high power image (40X). Thin black arrows point to examples of tissue injury (congestion of vessels).

**Fig 7:**
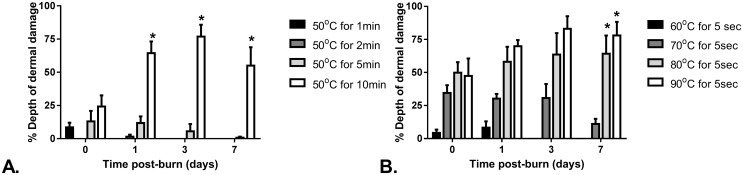
Depth of dermis damaged as a percentage of total dermal depth, for each condition and time point. A. 50°C burns, B. 5 sec exposure burns. Bars are mean ± SEM. * significantly deeper than other burn conditions at the same time point (p ≤ 0.001).

The macroscopic and histologic appearance of wounds was consistent between replicates of the same burn condition at each time point (Figs 6 and 7). By 7 days post-burn there was no microscopic evidence of cell damage for the 50°C for 1 and 2 min burns or the 60°C for 5 sec burns. All other burn conditions showed some degree of tissue damage at 7 days post-burn. A gradient of tissue injury was observed with more damage to the cells in the superficial dermis compared to the deeper dermal tissue below. Results for the line representing depth of damage are the deepest level to where damage was observed i.e. the endpoint of this graduated tissue damage.

For 50°C water, by 24hrs post-burn, the 10 min duration group showed significantly deeper tissue injury than all other exposure durations (p ≤ 0.0001), with damage depth observed to increase up to day 3. For 5 sec duration burns by day 7, the 80°C and 90°C scalds had significantly deeper dermal damage than the 70°C scalds (p ≤ 0.001). Extent of damage was also observed to increase over time post-burn for both the 80°C and 90°C scalds, this was statistically significant for 90°C for 5 sec scalds, showing deeper damage at day 3 and 7 post-burn compared to 1hr post-burn (p ≤ 0.02). Conversely, for 70°C scalds there was no increasing extent of damage over time post-burn and although it was not statistically significant, the extent of damage on day 7 was less than the damage at earlier time points.

A blinded re-test (n = 30) of randomly selected sections was performed by the same examiner, with a high intra-observer reliability rating (r = 0.87)

### 3.3 Subdermal temperature

To compensate for variations in the baseline subdermal temperatures from individual animals (34.2 ± 0.9°C), the results were calculated as change in temperature from starting temperature. The magnitude of subdermal temperature change was greater for burns of low temperature/long duration than for higher temperature/shorter duration burns. Maximum subdermal temperatures for each burn condition are given in Table 1. For short duration burns (5sec) the subdermal temperature continued to rise over the first 60 seconds post-burn, in contrast to the long duration burns (2 to 10 min) where the temperatures decreased immediately after heat source removal. The average distance from the bottom of the dermis to the subdermal temperature probe was 2.6 ± 0.6mm, measured with ultrasound *in vivo*.

**Table 1:** Subdermal temperatures for each burn condition.

Burn temperature (°C)/duration (sec)	Maximum subdermal temperature ± SEM (°C)	Maximum change in subdermal temperature ± SEM (°C)	Highest subdermal temperature recorded (°C)
50/60	38.6 ± 1.1	4.4 ± 1.3	44.0
50/120	41.4 ± 1	7.0 ± 1	43.6
50/300	41.8 ± 0.9	7.4 ± 1.1	45.5
50/600	43.9 ± 1	9.8 ± 1.3	47.8
60/5	35.9 ± 0.4	2.7 ± 0.8	36.5
70/5	35.9 ± 0.5	2.4 ± 0.2	37.0
80/5	37.2 ± 1.1	2.9 ± 0.9	39.0
90/5	39.1 ± 0.2	4.8 ± 0.6	39.5

### 3.4 Laser Doppler Imaging

**Fig 8:**
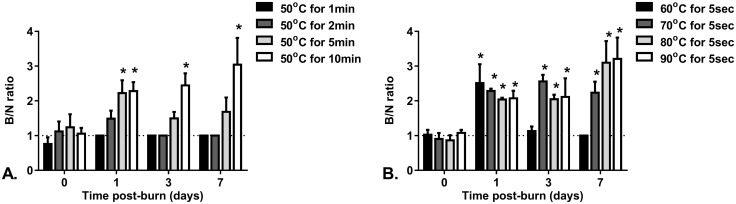
Laser Doppler Imaging Burn to Normal (B/N) ratio for different burn conditions. A. 50°C scalds and B. 5 sec scalds at different time points post-burn. Bars are mean ± SEM. *significantly different to normal (B/N = 1, p ≤ 0.05).

The mean perfusion units (PU) of normal tissue (perilesional unburned skin) was 238.4 ± 74.1. There was no significant difference in the mean PU of normal skin for scans performed on different days. The mean blood perfusion ratio of burned skin (B) to normal (N) perilesional skin for all burn conditions tested at each time point is shown in Fig 8. At 1hr post-burn there was no significant difference in the B/N ratio between un-burned normal skin and any of the burns. For 50°C water; at 24hrs post-burn the 5 and 10 min duration burns showed significantly higher B/N ratios than normal (p ≤ 0.05). However, by day 3 and 7 post-burn the only 50°C burn showing a higher than normal B/N ratio was the 10 min duration burn. For water applied for 5 sec; at 24hrs all the burns had significantly higher B/N ratios than normal (p ≤ 0.05). By day 3 and 7 all burns, except for the 60°C (which had no visible burn), showed significantly higher than normal B/N ratios. None of the burn conditions tested had a B/N ratio significantly lower than unburned skin at any of the scan time points. For both the 80°C and 90°C burns there was a strong trend of increasing perfusion with time post-burn, with B/N ratios at day 7 significantly higher than at 24hrs and day 3 (p ≤ 0.04, p ≤ 0.03).

## 4 Discussion

An effective burn model must have reliable and consistent tissue injury. One of the major advantages of a scald burn model is that the hot water is able to uniformly cover the entire area of exposed skin despite any irregularities in the skin surface. It has been reported for porcine contact burn models that less than optimal close contact between the burn infliction device (e.g. metal bar) and the skin surface [10, 33] can result in variability and inconsistencies in uniformity of depth of tissue injury. Researchers have attempted to overcome this effect by utilising a bottomless bottle sealed with malleable plastic wrap [15, 34, 35] which allows increased flexibility of the device to conform to the curved body contour of the flank. However even with this method, small irregularities in the skin surface or air pockets trapped between the device and the skin [36] may lead to variability in the uniformity of contact achieved. The consistency in the visual surface appearance of the scald burns immediately after creation confirms the uniformity of contact between the heat source (water) and the skin. In addition to consistency of surface appearance, it is arguably the uniformity of the depth of tissue injury (evaluated microscopically) for a specified burn condition which is required to verify the reliability of a burn model. The deepest level to where observable tissue damage was detected was consistent between replicates of the same burn condition with this model.

By taking biopsies at different time points in the early post-burn period (over 7 days) a more accurate assessment of the depth of tissue injury can be conducted. The dynamic phenomenon of burn wound progression has been well documented since it was described by Jackson [37]. As burn wounds evolve over time, caution is advised in interpreting early post-burn histological appearance. Here, by day 3 post-burn the full extent of the damage to the dermis was better recognised, as has been reported by others [37, 38]. For burns of moderate severity (80°C and 90°C for 5 sec and 50°C for 10 min) there was a general trend of increasing depth of damage observed from 1 hr post-burn to day 3 post-burn, when the deepest level of tissue damage was recorded. This indicates that biopsies taken in the first 24 hrs post-burn may underestimate the extent of tissue damage for some burn conditions, and should be interpreted with caution. Whether this delay in detecting the full extent of damage is as a result of the inability to identify functional cell damage with H&E staining or due to burn wound progression is unclear. Future work using alternative staining techniques, similar to those investigated by others using a contact burn model [30], will be performed to better assess cell viability.

The 70°C for 5 sec scalds were observed to show a similar depth of dermal damage from 1hr to day 3 post-burn. However, by day 7 these burns revealed histological evidence of healing and repair with regeneration of the epidermis and reduced depth of tissue damage. Comparatively, at day 7 the 80°C and 90°C for 5 sec scalds had indicators of both viability (epithelial proliferation, appearance of organising granulation tissue) and continued deep dermal tissue damage (inflammatory cell infiltration and endothelial cell damage), indicating that these burns were more severe. Whether these burns are representative of a severe deep partial thickness injury which takes longer than 3 weeks to heal and requires grafting is not established here, as time to healing for the different wound conditions was not investigated. Results from this study will be used to guide future investigations where a small range of burn conditions will be followed for longer than 7 days and time to healing established, so that histological indicators of damage can be correlated to clinical wound healing.

Clinically, it is recognised that deep dermal partial thickness burns require more extensive treatment than superficial dermal partial thickness injuries. Consequentially, there is significant interest in being able to predict the threshold temperature required to cause injury to the deep dermal tissue from a spill/splash exposure. While results presented here are for histological assessment of tissue damage and not time to healing, it is still meaningful to relate these findings to previous studies predicting burn injury depth in human skin. Here, a 5 second exposure time was considered to be representative of a spill/splash injury. Histological assessment at day 7 indicates that water ≥ 80°C resulted in injury to the deep dermal tissue. This injury threshold is at the lower end of those previously stated for numerical modelling [39, 40] and simulation [41] studies, where predictions for burn injury depth in human skin are reported. Additionally, our results concur with a recent study which quantified scald burns from hot beverage spills using both experimental data (obtained from skin and skin tissue surrogates) and numerical simulations [42, 43]. The authors reported a spill temperature of 82°C as the threshold for mid-dermal injury. We acknowledge, as do others [40, 42, 43], that experimental and numerical modelling studies represent an idealised situation and their limitations should be considered when comparing to the reality of human burns. The circumstances of each accidental spill/splash injury are unique and threshold for injury estimates reported here should be considered as a guide, rather than absolute.

Porcine burn models for investigating novel diagnostics and therapeutics are developed by creating a burn of pre-defined injury severity [14–16, 18, 29], which allows for assessment of healing progression. Presented here was an experimental model with emphasis placed on exploring the extent of tissue injury resulting from exposure to different scald burn conditions, similar to those encountered by children. As such, the deepest line of damage was representative of the deepest point at which any evidence for tissue damage was observed. For most of these scald burns, a gradient of tissue injury was observed (most apparent by day 3 and 7 post-burn), with more cells damaged in the superficial dermal tissue compared with the deeper dermis. For high temperature contact burns, a clear line of demarcation separates viable and non-viable areas within the dermis [12]. No such precise delineation was evident here and we suggest a gradient of injury is more prominent for scald burns of moderate severity (e.g. 80°C and 90°C for 5sec, 50°C for 10 min). Whether this is due to the differing pathophysiology of scald burns compared to contact burns, the magnitude of the heat dose applied or differences in the regenerative capacity of cells within the dermis becoming more apparent at these time points, is unknown.

The results shown in this study also illustrate the importance of comprehensive histological evaluation of depth of damage to the dermis for validation. Burn prevention strategies, such as legislation of hot water delivered to sanitary fixtures (e.g. bathrooms) to be no greater than 50°C, are based on Moritz and Henriques [11, 44] studies where the threshold for damage was described as full thickness destruction of the epidermis. However, as noted by others [45, 46], results from their studies have been and continue to be erroneously translated and extrapolated such that full thickness damage to the epidermis (trans-epidermal necrosis) is incorrectly equated to a more severe full thickness burn. For example, scald burn prevention literature states that exposure to 50°C water for 5 min causes a full thickness burn [47] and it only takes 5 to 6 sec for water at 60°C to cause a full thickness burn [48, 49]. Here, by day 7 the only 50°C burn showing any histological indicators for deep dermal damage was the 10 min exposure. By day 3 post-burn the 50°C for 5 min duration burns showed significant healing and were barely visible macroscopically with only minimal dermal damage detected histologically. Similarly, for the 60°C for 5 sec burn, by day 3 burns were not macroscopically visible and no dermal damage could be detected histologically. In future we will investigate a broader range of burn conditions, which will enable us to develop a clearer understanding of the relationship between burn depth and heat dose.

Measuring the subdermal temperature changes during thermal injury allows us to improve understanding of how heat is conducted through living skin. Quantitative analysis regarding heat conduction in skin using our experimental data and mathematical modelling is explored elsewhere [27]. In this study, the subdermal temperature profiles of the 60–90°C/5sec burns were similar, with a relatively small average increase in temperature from 2.7°C to 4.8°C and temperatures continued to rise after heat source removal. Conversely, we observed for longer durations of exposure (2 to 10 min) the magnitude of subdermal temperature change was much greater although the applied water temperature was lower (50°C). Interestingly, the change in subdermal temperature for the 50°C/1min burns and the 90°C/5sec burns was similar (4.4°C and 4.8°C respectively) however, these burns demonstrated markedly different tissue damage, with the 50°C/1min burn displaying no macroscopic or microscopic signs of injury at 24hrs post-burn, whereas the 90°C/5sec burns had microscopic evidence of damage to the deep dermis and a blanched white appearance indicative of a severe burn. Using clinically relevant burn conditions and relating temperature changes within the skin to tissue damage has significant clinical applications and requires further investigation. Future improvements to our model may include investigating how heat is transferred through the different layers of the skin by including multiple temperature measurements at different depths.

One of the most commonly cited limitations of a scald infliction method is the concern of increased risk of burn injury to the researchers when compared to contact burn models [6, 10]. We acknowledge there is potential for accidental burn injuries to investigators or the animal with our model, however no such incidents occurred. One key design feature unique to our model to safeguard against operator and unintentional animal injury was that only a small volume of water was in the device (just sufficient to cover the skin) at any given time. Furthermore our model reduces excess handling of the hot water by pumping the heated water straight from the water bath to the device rather than transfer via another heat source [12, 50]. The authors recommend additional personal protective equipment of long sleeved insulated rubber gloves and eyewear be worn for all high temperature scalds.

It is well documented in human studies that the reliability of LDI scanning in the immediate (early) post-burn period is questionable [51, 52]. Studies by Stetinsky *et al*. [51] report that the perfusion status of both superficial partial thickness and deep partial thickness burns were similarly low in the acute post-burn period (48 hours). They suggested that localised oedema may compress the vascular supply to the area and thus low perfusion reflects disruption rather than actual destruction of the microvasculature. Our results using a porcine model support this contention. Perfusion trends in the early (1 hour) post-burn period were generally lower than at all later time points with similar results recorded for all burns despite their differing severity (as determined by histopathology). However, in this study increased perfusion was observed by as early as 24 hours post-burn. Interestingly, none of the burn conditions tested displayed significantly lower than normal perfusion, including burns with evidence of damage into the deep dermis (measured histologically). Fourman *et al*. [53, 54] questions the reliability of LDI imaging to assess accuracy of predicting burn scarring [53] and viability of the zone of ischemia [54] using a porcine contact burn model. They observed difficulty in interpreting intermediate and low perfusion measurements, arguing that while LDI was able to reliably measure the hyperemic response of superficial dermal injuries it was less capable of differentiating deeper dermal porcine injuries. Results presented here support these claims, with a strong trend of increased perfusion from 24hrs post-burn for burns displaying superficial dermal damage. Additionally, burns considered to be of moderate severity (mid–dermal or with evidence of deep dermal damage e.g. 80–90°C/5sec, 50°C/10min) which we anticipated would show reduced blood flow (low B/N ratios) actually displayed significantly higher perfusion than normal by 24hrs. Nonetheless, for these burns the B/N ratio continued to increase to day 7 post-burn indicating some delay in reaching maximum perfusion compared to the other burn conditions tested in this study. It is possible higher than expected perfusion results may have occurred as a result of biopsies causing additional inflammation and pathological disruption to the burn injury area despite these areas being excluded from analysis. Future planned studies where healing outcomes are assessed will include LDI scans of burns where no prior biopsy samples have been taken.

Secondary outcome measures including analysis of severity of tissue injury with histology and LDI are presented here to demonstrate the models ability to create consistent burns and are intended to serve as a guide only when considering the exact heat dose required to create a burn of pre-defined injury severity for testing new burn treatments. Ultimately, we plan to use this model to better understand the relationship between burn injury severity and heat dose by investigating a broader range of burn conditions and including outcome measures such as time to healing.

In conclusion, whilst many investigators recognise both the relevance and advantages of a using a scald burn model over a contact burn model for certain studies, until now it has often been cited as too technically challenging and posing an unacceptable risk of injury to investigators. Presented here is a reliable and safe method of scald burn creation in a porcine model. Additionally the novel apparatus with continually refreshed water improves consistency of scald creation for long duration exposures.
